# Predicting chronic kidney disease progression with artificial intelligence

**DOI:** 10.1186/s12882-024-03545-7

**Published:** 2024-04-26

**Authors:** Mario A. Isaza-Ruget, Nancy Yomayusa, Camilo A. González, Catherine Alvarado H., Fabio A. de Oro V., Andrés Cely, Jossie Murcia, Abel Gonzalez-Velez, Adriana Robayo, Claudia C. Colmenares-Mejía, Andrea Castillo, María I. Conde

**Affiliations:** 1 Pathology and clinical laboratory. INPAC research group. Clinica Colsanitas. Keralty group, Fundación Universitaria Sanitas, Bogotá, Colombia; 2 Specialist in Internal Medicine and Nephrology, Keralty Global Institute of Clinical Excellence, Unisanitas Translational Research Group, Bogotá, Colombia; 3 Specialist in Internal Medicine and Nephrology, Unisanitas Translational Research Group. Renal Unit. Clinica Colsanitas, Bogotá, Colombia; 4 Clinical pathologist. Clinica Colsanitas, Bogotá, Colombia; 5 Internal Medicine resident, Fundación Universitaria Sanitas, Bogotá, Colombia; 6 Health Management Institute, Fundación Universitaria Sanitas, Bogotá, Colombia; 7 Adjunct Physician in Preventive Medicine and Public Health at the Maternal and Child, Insular University Hospital Complex, Las Palmas de Gran Canaria, Spain; 8 Specialist in Internal Medicine and Nephrology, Institute for Health Technology Assessment (IETS), Bogotá, Colombia; 9 Clinical Epidemiology, Research Unit. INPAC research group, Fundación Universitaria Sanitas, Bogotá, Colombia; 10 Evaluation and Knowledge Management. EPS Sanitas, Bogotá, Colombia; 11 Specialist in Medical Law and Global Health Diplomacy, MSc Public Health, EPS Sanitas, Bogotá, Colombia

**Keywords:** Renal insufficiency, Renal replacement therapy, Prediction, Artificial intelligence, Machine learning

## Abstract

**Background:**

The use of tools that allow estimation of the probability of progression of chronic kidney disease (CKD) to advanced stages has not yet achieved significant practical importance in clinical setting. This study aimed to develop and validate a machine learning-based model for predicting the need for renal replacement therapy (RRT) and disease progression for patients with stage 3–5 CKD.

**Methods:**

This was a retrospective, closed cohort, observational study. Patients with CKD affiliated with a private insurer with five-year follow-up data were selected. Demographic, clinical, and laboratory variables were included, and the models were developed based on machine learning methods. The outcomes were CKD progression, a significant decrease in the estimated glomerular filtration rate (eGFR), and the need for RRT.

**Results:**

Three prediction models were developed—Model 1 (risk at 4.5 years, *n* = 1446) with a F1 of 0.82, 0.53, and 0.55 for RRT, stage progression, and reduction in the eGFR, respectively,— Model 2 (time- to-event, *n* = 2143) with a C-index of 0.89, 0.67, and 0.67 for RRT, stage progression, reduction in the eGFR, respectively, and Model 3 (reduced Model 2) with C-index = 0.68, 0.68 and 0.88, for RRT, stage progression, reduction in the eGFR, respectively.

**Conclusion:**

The time-to-event model performed well in predicting the three outcomes of CKD progression at five years. This model can be useful for predicting the onset and time of occurrence of the outcomes of interest in the population with established CKD.

**Supplementary Information:**

The online version contains supplementary material available at 10.1186/s12882-024-03545-7.

## Introduction

Chronic Kidney Disease (CKD) is a global public health problem [1–3]. In 2017, the global prevalence was 9.1%, with approximately 700 million cases [4]. In Colombia, it has been estimated a gross and underdiagnosed prevalence of 1.54 CKD cases per 100 inhabitants in 2022 [5]. Primary and secondary prevention are the mainstay of treatment, and management according to the risk profile is a priority. However, according to the United States Renal Data System (USRDS) report, approximately 35.4% of patients with CKD are referred late to interdisciplinary programs, probably due to failure in adequate risk profile classification [6, 7]. During the last decade, several prediction models to estimate the probability of CKD progression, with predictors such as age, estimated glomerular filtration rate (eGFR), serum albumin level, and the presence of comorbidities, are being used. These models usually predict CKD progression through eGFR loss or need for RRT (dialysis or kidney transplant).[8] However, the models available show some limitations, such as lack of estimation of the competing risk of death or non-fatal cardiovascular disease associated with disease progression [9], lack of validation in reference centers for patients with CKD, significant predominance of an ethnic group in the cohorts used [10], and low rates of outcomes such as need of RRT [11]. Prediction models are usually built using logistic regression or Cox proportional hazards, and recently, some have used artificial intelligence methods, such as neural networks and random forests, among others [12].

One of the most validated models around the world, ESKD, have an excellent performance, with a C statistic of 0.917 (95% confidence interval [CI], 0.901–0.933; *P* < 0.001) in the development cohort, and 0.841 (95% CI, 0.825–0.857) in the validation cohort [8, 9]. No predictive models have been developed or validated in Colombia. The primary rationale for developing a new model in our context stems from the fact that current prediction models include variables present in fewer than 50% of our patients (such as albuminuria, phosphorus, bicarbonate). [13] These are often substituted with qualitative measures of proteinuria, proteinuria in a 24-hour urine sample, or indirectly with lipid profiles or blood albumin levels. [5] Constructing a model based on the available variables is imperative for our country and may be applicable to other low-income countries. Therefore, this study aimed to develop and validate a predictive model for the risk of progression and the need for RRT in patients with stage 3–5 CKD, which may be clinically useful for assisting our country’s healthcare system.

## Methods

### Design and population

This was a retrospective, closed cohort, observational study. The cohort was selected based on the electronic medical records and clinical laboratory data of patients affiliated with a private health insurer in Colombia (EPS Sanitas). This register correspond to all patients treated for chronic kidney disease. In the year of selection of the cohort, it had approximately 2.5 million members and a geographical distribution mostly represented by the country’s capital, with 48.5% of the population, followed by the central and northern regions with 13.8% and 12.4%, respectively, including patients with CKD both in primary care units as well as in high complexity centers.

Patients with a confirmed diagnosis of CKD in stages 3–5 were included, and a 5- year follow-up from 2013 to 2018 was implemented. Confirmed diagnosis of CKD was defined as a decrease in eGFR (between 60 and 15 mL/min/1.73 m2) or albuminuria (24-h urine albumin excretion rate > 30 mg/24 h or urine albumin/creatinine ratio > 30 mg/g [3 mg/mmol]) of any etiology for more than 3 months. eGFR was calculated using the Chronic Kidney Disease Epidemiology Collaboration (CKD-EPI) 2009 formula based on serum creatinine, sex, and the age of the research subject.13 Pregnant patients and patients who met the clinical criteria for starting RRT (dialysis or transplant) or those who were already receiving it at the start of follow-up were excluded.

### Outcomes

The outcomes evaluated were as follows: (1) CKD progression: defined as progression in the stages of this condition based on a decrease in the eGFR; (2) significant decrease in the eGFR: defined as a progression to greater than 25% reduction in the eGFR; and (3) indication for RRT: defined as the need for dialysis and/or kidney transplant.

### Predictors

The predictors that were initially considered according to the importance reported in the literature are shown in the supplementary data (Supplementary data, Table S1). Physical examination and laboratory test predictors were measured within a time window of ± 120 days with respect to the initial eGFR. Predictors were selected through a heuristic factor; based on information availability for at least 50% of the patients, the combination of these factors that keeps the greatest number of patients is studied and selected. This approach lies mainly in the availability of information on clinical results and rules out the use of data imputation due to confidence intervals could be artificially narrow. Predictors based on medical history (comorbidities) were identified when recorded on previous dates or on the same date in which the eGFR was obtained, and cases in which their incidence in the samples was less than 1% were excluded.

Three predictive models were developed using two different statistical approaches: Model 1, risk analysis at 4.5 years; Model 2, time-to-event analysis; and Model 3, time-to-event analysis with reduced variables. Model 3 corresponds to a reduced version of Model 2 predictors, which were obtained after implementing a validation in an external cohort.

### Population size and selection

**Fig. 1:**
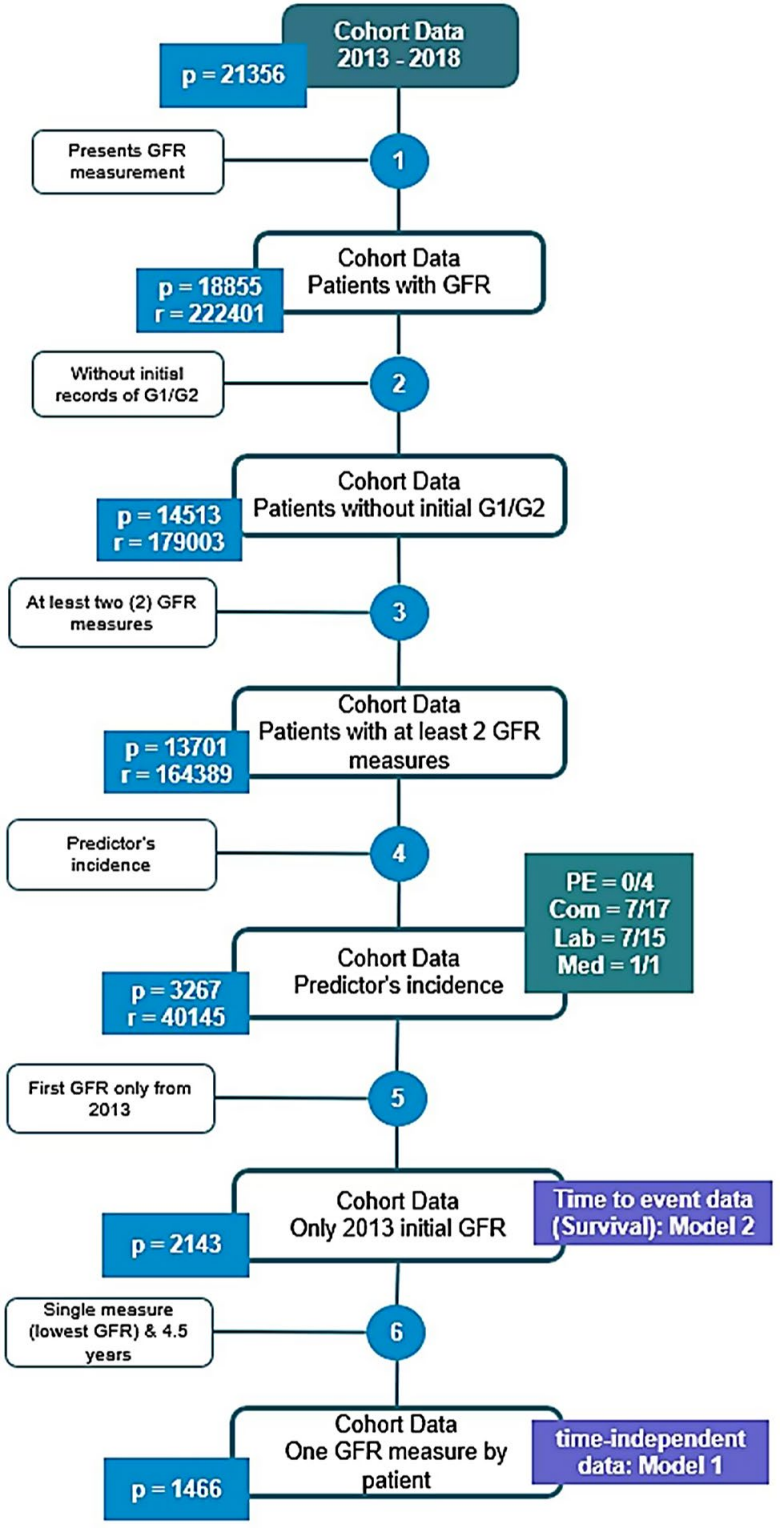
Selection of the population for Models 1 and 2

Patients with at least one eGFR record of less than 60 mL/min/1.73 m2 were selected from a sample of 21,356 patients with a confirmed diagnosis of CKD identified in the institutional registry (Fig. 1). Next, the presence of at least two eGFR measurements during follow-up and the availability of complete data in relation to the selected predictors were verified. For Model 1, patients with a eGFR measurement in the first year of follow-up (the lowest value) and at 4.5 years were selected, with a final population of 1,466 patients. For Models 2 and 3, patients with the first eGFR measurement in 2013 (beginning of the cohort) were included, for a total of 2,143 subjects. Sensitivity analyses for clinical and laboratory predictors were performed for each model (Supplementary data, Tables S1 and S3).

### Model development and validation

The following two strategies were used for the selection of predictors: (1) A review of the linear correlation between the same predictors and (2) An evaluation of the level of importance for prediction of each one through an engineering process using the Recursive Feature Elimination (RFE) algorithm. The coefficients of each predictor and subsets of predictors with optimal performance were estimated for each outcome of interest. Performance, according to this algorithm, was estimated with the accuracy metric. No imputation procedure for missing data was performed. Qualitative variables with multiple categories were classified into subgroups of dichotomous predictors before this process, following the dummy variables strategy for optimal training of the base algorithm.

The predictors included in Models 1 and 2 were age, sex, marital status, geographical region of residence, socioeconomic status, initial eGFR in mL/min/1.73 m2, presence of diabetes mellitus, coronary heart disease, arterial hypertension, anemia, heart failure, cerebrovascular disease, rheumatoid arthritis, Non-steroidal anti-inflammatory drugs (NSAID) consumption, creatinine levels (mg/dL), hemoglobin (gr/dL), serum potassium (mEq/L), HDL cholesterol (mg/dL), LDL cholesterol (mg/dL), and triglycerides (mg/dL). In the case of Model 3, only age, sex, region of residence, diabetes mellitus, arterial hypertension, hemoglobin, creatinine, HDL cholesterol, LDL cholesterol, and baseline eGFR were considered because the external validation cohort relied only on these predictors.

Baseline eGFR was estimated taking as reference the minimum value among the measurements recorded in 2013. This same strategy was used to identify the final eGFR; we recorded the minimum measurement within a range of ± 120 days of the date corresponding to 4.5 years from the baseline eGFR. Qualitative variables are presented as absolute and relative frequencies. Quantitative variables are reported as medians and interquartile ranges (IQR).

#### Model 1

Models were generated for each outcome using the following three methods: logistic regression and two other methods based on machine learning called Neural Networks (NN) and Random Forests (RF). A black box method was used for the neural network model, which was robust for complex data patterns. Their performance was evaluated individually once the resulting models were generated and compared using F1, accuracy, sensitivity, and precision metrics.

#### Model 2

A follow-up mechanism (in months) was initially designed to establish the risk of appearance of the three outcomes of interest over time. For the outcomes, significant decrease in eGFR and CKD stage progression, the first eGFR measurement that showed this deterioration, or change in stage was selected. For the RRT outcome, the need to start replacement therapy was determined as the end point of follow-up. Three artificial intelligence methods were used for modeling: Cox Penalty (P-Cox), Random Survival Forest (RSF), and Gradient Boosting Model (GBM). The concordance index metric (C-index) was used to compare the results of these three approaches.

#### Model 3

As in model 2, the same follow-up design was used for the three outcomes. The same three survival models were estimated: P-Cox, RSF, and GBM. Furthermore, based on the purpose of this reduced model, an external validation was performed for a fully audited cohort, extracted using data from the Sanitas Renal Unit (URS) between 2019 and 2021. This validation cohort included 648 patients. (Supplementary data, Figure S1). The sample was divided into the following two groups for implementation and evaluation of the models: training (70%) and test (30%), and a cross-validation process was performed on the training set.

### Development of the electronic calculator

Based on the model obtained, an electronic calculator with a graphical interface was developed on a web platform using the Extreme Programming software development process model for clinical use.

Model 3 was chosen to develop an electronic risk calculator given the optimal validation results and the data completeness challenges in electronic health records. Three endpoints (API) were formulated for each outcome (CKD progression, significant decrease in the eGFR, and RRT). These subroutines were integrated into a RESTful service, providing a response that includes the C-index model performance, follow-up time periods risk probabilities, and an encoded image for the survival curve. This information is retrieved and presented in the electronic calculator for clinical use.

## Results

### Model 1

The sociodemographic, clinical, and laboratory characteristics of the Model 1 cohort are shown in Table 1. There was a high proportion of female subjects, more than 90% of the population corresponded to the middle stratum, and the region of origin with the highest proportion corresponded to the country’s capital. The most frequent clinical history variables were the presence of arterial hypertension, consumption of NSAIDs, and diabetes mellitus.

**Table 1:** Sociodemographic, clinical, and laboratory characteristics for the population in Model 1

Predictor	All	Stage Progression	Decrease in eGFR	RRT
	*N* = 1466	*n* = 386	*n* = 268	*n* = 56
**Sex** (%F)	59.70%	52.07%	51.12%	42.86%
**Stratum**				
Low (1–2) %	4.42%	4.40%	4.47%	1.78%
Mid-level (3–4) %	90.61%	89.38%	88.44%	94.65%
High (5–6) %	4.97%	6.22%	7.09%	3.57%
**Region** Bogotá %	63.64%	65.54%	65.29%	71.44%
Eastern %	5.52%	6.99%	6.71%	10.71%
Central %	13.17%	8.55%	6.35%	7.14%
Pacific %	11.36%	12.44%	15.30%	3.57%
Caribbean %	6.23%	6.48%	6.35%	7.14%
Other	0.08%	0%	0%	0%
**Marital Status**				
Married %	45.35%	43.26%	41.42%	53.57%
Unmarried %	54.65%	56.74%	58.58%	46.43%
**Age (IQR)**	77 (72–81)	77 (71–81)	76 (71–80)	66 (57–73)
**Lab tests (IQR)**				
eGFR mL/min/1.73 m2	48.78 (40.18–55.20)	47.11 (37.22–53.02)	43.34 (32.44–53.01)	22.62 (15.53–30.33)
Triglycerides mg/dL	133.95 (102–180.12)	138.2 (104–192.72)	134.1 (105–191.42)	162.1 (112.57–239.32)
Hemoglobin gr/dL	14 [13–15]	14.1 (12.8–15.1)	13.9 (12.5–15.1)	12.35 (11.07–14.5)
Creatinine mg/dL	1.24 (1.05–1.47)	1.32 (1.11–1.54)	1.36 (1.16–1.84)	2.58 (1.99–3.22)
Potassium mg/dL	4.52 (4.23–4.88)	4.58 (4.24–4.96)	4.61 (4.22–5.01)	4.93 (4.42–5.35)
Cholesterol HDL mg/dL	49.95 (40.79–60.1)	48.3 (39.4–59.3)	47.65 (38.87–56.8)	43.75 (36.41–52.57)
Cholesterol LDL mg/dL	102.21 (79.19–127.53)	102.68 (77.04–126.05)	97.58 (73.75–124.07)	99.28 (75.49–132.49)
**Comorbidities**				
Diabetes	24.68%	31.60%	36.19%	44.64%
Hypertension	92.90%	88.08%	87.31%	75%
Anemia	3.85%	4.40%	5.22%	3.57%
Coronary disease.	18.14%	20.72%	22.39%	16.07%
Cerebrovascular disease	1.42%	-	-	0%
Heart failure	15.06%	17.87	20.15%	12.5%
Rheumatoid arthritis	1.49%	-	-	-
Consumption of NSAIDs	28.08%	23.83%	20.52%	17.85%

eGFR: Glomerular Filtration Rate. F: Feminine. NSAIDs: Non-steroidal anti-inflammatory drugs

The cumulative incidence for the outcome of stage progression was 26.3% (95% CI 24.2–28.7); for decreased eGFR was 18.3% (95% CI 16.4–20.4); and for RRT was 3.8% (95% CI 2.9–4.9). The bivariate analysis between predictors and outcomes is shown in the supplementary data (Tables S4, S5 for Model 1; Tables S6, S7 for Model 2).

**Fig. 2:**
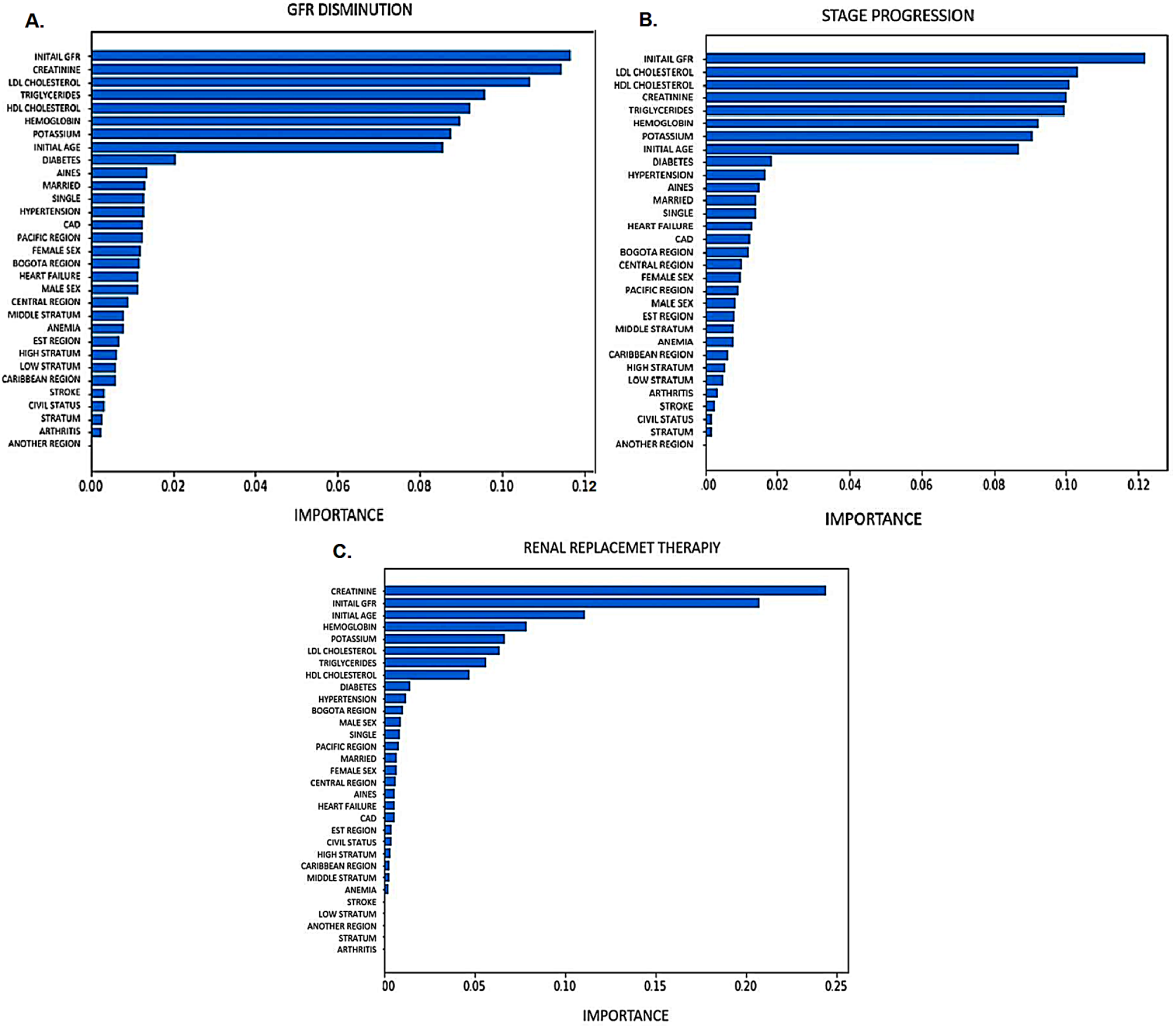
Predictors in order of importance for each outcome according to the recursive feature elimination method

The selection of predictors according to the RFE algorithm for each outcome is shown in Fig. 2. The most relevant predictors were age, initial eGFR, creatinine, LDL, triglycerides, HDL, hemoglobin, potassium, and history of diabetes mellitus or arterial hypertension, especially for the outcomes of significant decrease in eGFR and stage progression.

The performance of the different models for risk prediction at 4.5 years based on artificial intelligence metrics are shown in Table 3. The NN and RF models showed a similar performance according to F1 for the estimation of significant decrease in eGFR and stage progression. Logistic regression showed better performance than NN and RF for the RRT.

### Model 2

**Fig. 3:**
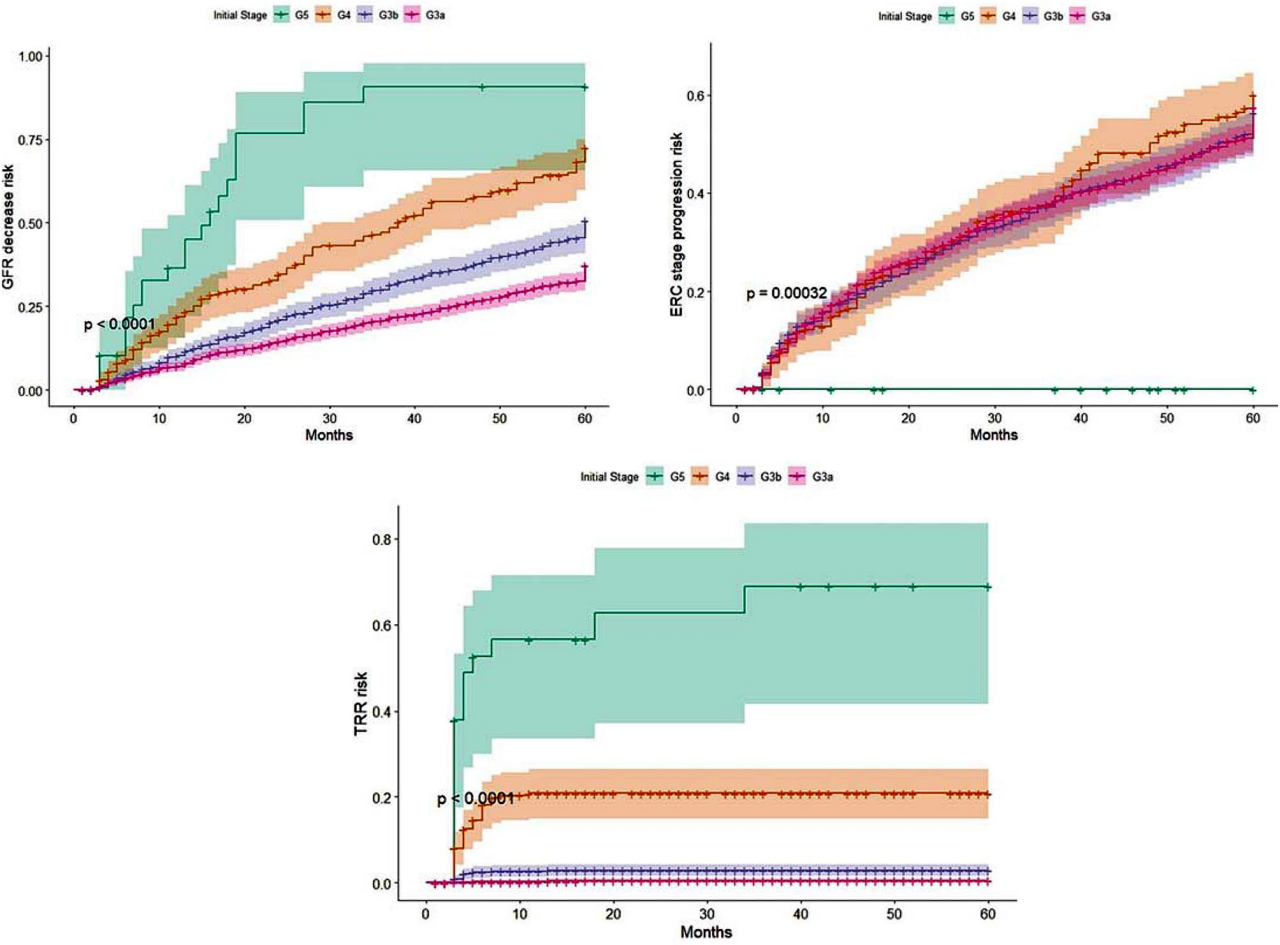
Survival curves for outcomes according to Model 2

The main sociodemographic, clinical, and laboratory characteristics of the time-to- event analysis model cohort are shown in Table 2. The 5-year incidence rate for stage progression was 48.3% (95% CI 46.2–50.5); for decreased eGFR was 37.4% (95% CI 35.4–39.5); and for RRT was 3.9% (95% CI 3.1–4.8). The survival curves according to the initial eGFR stage for the outcomes of interest are shown in Fig. 3. The probability of RRT was higher for subjects with stage 4 and 5 CKD. The performance of the estimated models for prediction based on time-to-event analysis is presented in Table 4. The model with the best performance for prediction of time-to-event was RSF according to the C-index. For RRT and significant decrease in eGFR, the three algorithms showed a similar performance, while for stage progression, the GBM showed the best performance.

**Table 2:** Sociodemographic, clinical, and laboratory characteristics for the population in Model 2

Predictor	All	Stage Progression	Decrease in eGFR	RRT
	*N* = 2143	*n* = 1035	*n* = 802	*n* = 83
**Sex** (%F)	52.97%	53.43%	51.12%	44.58%
**Stratum**				
Low (1–2) %	3.94%	4.63%	5.11%	1.21%
Mid-level (3–4) %	91.41%	90.16%	89.65%	95.18%
High (5–6) %	4.65%	5.21%	5.24%	3.61%
**Region** Bogotá %	67.92%	63.19%	65.46%	66.26%
Eastern %	6.59%	6.86%	6.36%	9.64%
Central %	9.86%	12.66%	9.85%	12.05%
Pacific %	10.56%	12.17%	13.22%	6.03%
Caribbean %	5.07%	5.12%	4.99%	6.02%
Other	0.06%	0%	0.12%	0%
**Marital Status** Married %	45.44%	45.22%	42.77%	55.42%
Unmarried %	54.66%	54.88%	57.33%	44.58%
**Age (IQR)**	77 (71–82)	78 (73–83)	78 (73–83)	70 (58–75)
**Lab tests (IQR)**				
TFG mL/min/1.73 m2	44.97 (35.25–52.93)	47.31 (37.01–52.68)	44.90 (34.46–52.58)	22.35 (15.52–32.31)
Triglycerides mg /dL	134.0 (100.5–182.4)	134.4 (100.1–185.05)	134.35 (101.80–184.75)	147.8 (109.85–211.45)
Hemoglobin gr/dL	13.9 (12.7–15.0)	13.9 (12.7–15.01)	13.8 (12.6–14.97)	12.6 (11.35–14.5)
Creatinine mg/dL	1.33 (1.13–1.67)	1.29 (1.09–1.55)	1.33 (1.13–1.68)	0 (0–26)
Potassium mg/dL	4.58 (4.26–4.97)	4.56 (4.26–4.93)	4.61 (4.28–4.98)	4.91 (4.43–5.35)
Cholesterol HDL mg/dL	48.4 (39.5–58.7)	48.4 (39.55–59.25)	47.5 (38.83–58.28)	45.7 (37.45–53.4)
Cholesterol LDL mg/dL	99.1 (74.1–125.1)	101.68 (76.95–126.98)	98.48 (73.95–124.27)	101.66 (74.72–131.17)
**Comorbidities**				
Diabetes	24.42%	23.38%	24.94%	34.94%
Hypertension	80.58%	77.29%	74.31%	67.47%
Anemia	4.15%	3.77%	3.61%	2.4%
Coronary disease	19.74%	15.84%	17.95%	14.46%
Cerebrovascular disease	1.60%	-	-	1.21%
Heart failure	20.18%	17.49%	19.95%	12.05%
Rheumatoid arthritis	1.99%	-	-	-
Consumption of NSAIDs	21.80%	21.83%	19.2%	13.25%

eGFR: Glomerular Filtration Rate. F: Feminine. NSAIDs: Non-steroidal anti-inflammatory drugs

**Table 3:** Performance of Model 1 according to the prediction algorithm

Outcome	Sensitivity	Accuracy	F1	Precision
**Renal replace therapy**				
Logistic Regression	0.79	0.97	0.82	0. 85
Neural Network	0.61	0.95	0.64	0.70
Random Forest	0.62	0.96	0.67	0.88
**Stage Progression**				
Logistic Regression	0.54	0.67	0.52	0.57
Neural Network	0.53	0.68	0.51	0.57
Random Forest	0.54	0.69	0.53	0.60
**eGFR Progression**				
Logistic Regression	0.55	0.78	0.55	0.64
Neural Network	0.53	0.78	0.51	0.62
Random Forest	0.53	0.79	0.51	0.65

**Table 4:** Performance of time-to-event models for the outcomes of interest

Model	Stage Progression	eGFR Progression (significant reduction)	RRT
**P-COX**			
**Test**	0.5790	0.6297	0.9082
**Random survival forest**			
**Test**	0.6650	0.6759	0.8926
**Gradient boosted model**			
**Test**	0.6733	0.6701	0.8953

Test: C- Statistic concordance Index, eGFR: Glomerular Filtration Rate, P-cox: Penalization cox, RRT: Renal replacement Therapy

### Model 3

For this model, the sociodemographic, clinical, and laboratory characteristics were identical to those of the time-to-event analysis model cohort (Table 2), but omitting the predictors of marital status, socioeconomic status, coronary heart disease, anemia, heart failure, cerebrovascular disease, consumption of NSAIDs, serum potassium, and triglycerides.

The training results for the three estimated models are shown in Table 5. The GBM showed slightly better performance than the other models for all three outcomes. It was determined that the RRT outcome showed better results than the other outcomes in the three models.

**Table 5:** Performance of reduced time-to-event models for the outcomes of interest

Model	Stage Progression	eGFR Progression (significant reduction)	RRT
**P-COX**			
**Test**	0.5648	0.6917	0.8603
**Random survival forest**			
**Test**	0.6713	0.6807	0.8603
**Gradient boosted model**			
**Test**	0.6874	0.6847	0.8887

Test: C- Statistic concordance Index, eGFR: Glomerular Filtration Rate, P-cox: Penalization cox, RRT: Renal Replacement Therapy

Regarding the external validation with the URS cohort, the results of the C-index metric for the different models reduced from the validation data, compared against the test results (20% of 2,143 patients) from the initial cohort, are shown in Table 6. In this case, the GBM algorithm performed better for the outcomes of significant decrease in eGFR and RRT. In contrast, the RSF algorithm was slightly better than GBM in terms of CKD progression.

**Table 6:** External validation performance and test of the reduced time- to-event models for the outcomes of interest

Model	Stage Progression	eGFR Progression (significant reduction)	RRT
P-COX			
**Test**	0.5648	0.6917	0.8603
Validation cohort	0.4938	0.5980	0.8284
**Random survival forest**			
**Test**	0.6713	0.6807	0.8603
Validation cohort	0.6664	0.6092	0.9307
**Gradient boosted model**			
**Test**	0.6874	0.6847	0.8887
Validation cohort	0.6926	0.5981	0.9518

Test: C- Statistic concordance Index, eGFR: Glomerular Filtration Rate, P-cox: Penalization cox, RRT: Renal Replacement Therapy

## Discussion

A model for predicting the progression of CKD to advanced stages and the need for RRT was developed and validated in this study. Risk at 4.5 years and time-to-event (survival analysis) in patients with CKD stages 3–5 were assessed based on data analysis using artificial intelligence tools. The proposed model showed adequate performance, allowing its systematic implementation in CKD clinical management programs, and guaranteeing its usefulness as part of the strategies that guide clinical decision making.

Publications of prediction models for chronic kidney disease have increased in recent years, and this has been analyzed in four recently published systematic reviews [14–17].However, skepticism still remains among clinicians regarding the performance and applicability of these models, apart from the fact that clinical practice standards are generic when it comes to defining a precise recommendation on this subject. For example, KDIGO recommends using prediction models for timely referral to RRT. However, it does not define how and when to use these tools [18].

In this context, it is important to have prediction tools that guide decision making, the management of prevention programs, and timely multidisciplinary intervention strategies. However, several of the published models include populations with different levels of disease severity and do not precisely define the outcomes and the time of disease progression in which they should be used [14]. Our study developed a prediction model that can be applied in patients with CKD stages 3–5, with a 5-year follow-up, determining the following as main outcomes: (i) progression of CKD stage based on the eGFR (ii) reduction greater than 25% in the eGFR compared to baseline, and (iii) onset of RRT (dialysis for more than 3 months or kidney transplant).

To minimize the risk of bias, a cohort of 2,143 patients distributed in three arms was included for each outcome. For the incidence of RRT, the entire cohort of 2,143 patients was included. For progression of CKD stage and significant decrease in eGFR, there were 2,060 patients, of which 1,035 and 802 presented the respective outcomes. Furthermore, the external validation process included a cohort of 648 patients. As it became evident during the cohort selection process, when working with real data, this study was contingent upon missing information, as is the case of alkaline phosphatase, where no data was found for 92.6% of the patients. Because these are precise clinical data, the use of imputation strategies was ruled out. On the other hand, as demonstrated by the performance metrics, the models that predict RRT could be showing data overfitting due to imbalance in the classes; there were very few patients who presented the outcome. This was especially evident in the external validation for RRT, where a C-index of 0.9518 was obtained, but this cohort only had two patients who showed this outcome.

The inclusion of a validation cohort increases the reliability of the models. This is how the kidney failure risk equation (KFRE) developed by Tangri et al. [7] has become the standard for comparison, given that the model has shown consistently good performance in patients with CKD stages 3–5 in several external validation studies with a low risk of bias. One of these validations included 31 multinational cohorts with a mean baseline eGFR of 46 mL/min/1.73 m2 and showed that the KFRE model has a high discrimination capacity and adequate calibration [15]. Another model validated with an external cohort and a low risk of bias is the Kaiser Permanente Northwest (KPNW) model [9].

Although some clear prognostic factors of this and other prediction models are the main ingredients of our model, the context of our country requires particular sociodemographic factors that are included here. Our cohort is consistent with the country’s reality, with an absence of relevant variables that limit the sample, and with greater participation of patients from the central region. Upon admission to the cohort, the largest participation included stage 3 patients, and the frequency RRT initiation decreased over time.

The prediction of CKD progression to advanced stages or admission to dialysis allows for the implementation of strategies for individualized treatment, control of risk factors, evaluation of population indicators, establishment of education management strategies, selection of renal support therapies, and preemptive renal transplantation [19].

Conventionally, risk factors related to CKD progression included in most prediction models have been demographic variables such as age, sex, and geographic origin, and clinical variables such as comorbidities, eGFR or its deterioration in the previous year, albuminuria, serum bicarbonate, albumin, calcium, hemoglobin, and phosphorus, among others [6].In recent publications describing other models proposed concomitantly with our own model, we found unconventional predictors such as biomarkers (CXCL12, NT-proBNP, NGAL, and troponin T) and the application of artificial intelligence methods [20–22].

Although CKD management programs in Colombia seem to be very well structured, they do not offer sub-specialized management programs for the entire population with CKD in terms of health policies. This is due to several reasons, in particular, a disproportion between the number of patients with CKD and the current number of nephrologists in the country. In their daily roles, these nephrologists cover different areas of work, such as critical care, hospital nephrology, kidney units, transplant groups, and outpatient and preventive services. This reveals an insufficient human resource, far from international standards (a nephrologist–patient ratio of approximately 1:2000 patients), requiring the support of non-nephrologists, trained and familiar with this group of patients. In general, these programs are configured based on an initial snapshot of the stage of the disease exclusively on the eGFR. It is possible to optimize the care of these patients with the implementation of the prediction models. Thus, a possible scenario can be proposed in which the care of stage 3 patients with a low risk of progression could continue in first level centers, improving the window of opportunity for the highly specialized care of patients classified showing a high risk of progression. This would lead to an improvement in cost-effectiveness indicators.

Among the strengths of our model, we can highlight the following: (1) the creation of a machine learning-based technological tool that makes it possible for non- specialized healthcare personnel to estimate the risk for patients. This is useful in prevention programs in the context of a limited specialized human resource; (2) the estimation of the risk of a significant decrease in eGFR as an additional outcome to stage progression benefits the early implementation of prevention strategies to avoid the deterioration of renal function, even within the same risk category; (3) the predictors were obtained from frequent registration data in the clinical follow-up records of this group of patients. The use of additional resources was not necessary for their application; (4) to date, no models have been developed in a population with sociodemographic characteristics like ours. This represents an opportunity to use a tool based on the context of our country and others in the Latin American region; (5) an external validation cohort was included, and the tool is designed to be implemented in a specific population of patients with stage 3–5 CKD; and (6) the translation of a mathematical model into a digital tool in the form of a calculator with a graphical, easy-to-use interface facilitates its use in these patient care scenarios.

This model has certain limitations as follows: (1) the follow-up period was 5 years, which limits the analysis of disease progression in some groups of patients; (2) there was no differentiation into racial groups, which affects the analysis of subgroups and progression according to this predictor, which is part of the tools frequently used to calculate the eGFR; (3) model development was based on a retrospective cohort, which reduced the availability of complete data in relation to the predictors of interest for the final analysis; (4) the population subgroup belonging to the middle-income socioeconomic stratum was overrepresented (90%), which is well above the percentages recorded in national evaluation surveys (45.5%) and which may imply more favorable socioeconomic contexts and determinants than those of the average Colombian population; 6) the percentage of representation of women was greater than 50%, which may not be related to the proportion of the population on dialysis or subjected to kidney transplant; and 7) the geographical distribution of the patients included in the sample was linked to the distribution of affiliates of the selected insurer, which does not have members in all regions of the country. This prevents the generalization of estimates to the entire national territory.

In conclusion, the developed model constitutes a tool to help manage the progression of CKD in terms of early intervention and optimization of available human resources.

### Electronic supplementary material

Below is the link to the electronic supplementary material.

Supplementary Material 1

## Data Availability

The datasets used and/or analyzed during the current study are available from the corresponding author on reasonable request.
