# Supplementary material for: Predicting chronic kidney disease progression with artificial intelligence
Source: BMC Nephrol. 2024 Apr 26;25:148. doi: 10.1186/s12882-024-03545-7 (PMC11055348; doi:10.1186/s12882-024-03545-7)
Supplement: Supplementary file 1 — Supplementary Material 1 [file 12882_2024_3545_MOESM1_ESM.doc]

SUPPLEMENTARY MATERIAL

PREDICTING CHRONIC KIDNEY DISEASE PROGRESSION WITH ARTIFICIAL INTELLIGENCE

Mario A. Isaza-Ruget^1^, Nancy Yomayusa^2^, Camilo A. González^3^, Catherine Alvarado H.4, Fabio A. de Oro V.5, Andrés Cely^6^, Jossie Murcia^7^, Abel Gonzalez-Velez^8^, Adriana Robayo^9^, Claudia C. Colmenares-Mejía^10^, Andrea Castillo^11^, María I. Conde^12^

# SUPPLEMENTARY MATERIAL

Table S1 Predictors that were initially considered according to the importance reported in the literature.

Table S2 and Table S3. Statistical Sensitivity Analyses of Clinical and Laboratory Predictors for each model.

Figure S1. External Validation Cohort Selection Process.

Table S4. External Cohort description

Bivariate Analysis between Predictors and Outcomes of Interest for Model 1 (Table S5 and Table S6) and Model 2 (Table S7 and Table S8).

Figure S2.

Figure S2. Calibration plots for Model 1

Supplementary data are available online.

TABLE S1. Predictors that were initially considered according to the importance reported in the literature.

| **Sociodemographic variables** | **Comorbidities** | **Laboratory Test** | **Physical examination**  **variables** |
| --- | --- | --- | --- |
| Age. Sex. Race.  Educational level Socioeconomic. status. | estimated GFR Diabetes Hypertension Acute kidney Injury  Current or previous smoking Urinary tract abnormality. Anemia.  Diabetic Retinopathy. Coronary Artery disease.  Heart failure. Peripheral arterial disease. Cerebrovascular disease.  Hepatitis B/C. HIV infection. Urolithiasis.  Rheumatoid. arthritis.  Systemic lupus erythematosus. Metabolic syndrome, NSAIDs  Family history of kidney disease exposure to heavy metals or agrochemicals | Creatinine. Sodium.  Potassium. Hemoglobin. Calcium.  Phosphorus. Serum albumin. Alkaline phosphatase.  Chloride. Bicarbonate. Albumin-to- creatinine ratio (ACR).  Albumin excretion rate (AER).  Total cholesterol. High-density lipoprotein cholesterol (HDL). Low-density lipoprotein cholesterol (LDL). Triglycerides.  HbA1c and C- reactive protein). | Weight .  Blood pressure. |

**GFR: Glomerular filtration rate, NSAIDs: Non steroidal anti inflammatory drugs.**

TABLE S2. Sensitivity analysis Model 1.

| **Variables** | **Not included**  **(n=12235)** | **Included in Model 1**  **(n=1466)** | **P-**  **value** |
| --- | --- | --- | --- |
| **Sociodemographic** | | | |
| Age | 78 (72–84) | 77 (72–81) | <0.001 |
| Sex | F: 7536 (61.6%) | F: 901 (61.5%) | 0.203 |
| Stratum | Mid-Level: 11329 (92.6%) | Mid-Level: 1322 (90.2%) | 0.012 |
| Region | Bogotá: 7879 (64.4%) | Bogotá: 932 (63.6%) | <0.001 |
| Marital status | Unmarried: 6839 (55.9) | Unmarried: 785 (53.6%) | 0.287 |
| **Comorbidities** | | | |
| Initial stage | G3a: 8760 (71.6%) | G3a: 896 (61.1%) | <0.001 |
|  | G3b: 2655 (21.7%) | G3b: 437 (29.8%) | <0.001 |
|  | G4: 722 (5.9%) | G4: 117 (8.0%) | <0.001 |
|  | G5: 98 (0.8%) | G5: 16 (1.1%) | <0.001 |
| Diabetes | Yes: 2544 (20.8%) | Yes: 362 (24.7%) | 0.001 |
| Hypertension | Yes: 9861 (80.6%) | Yes: 1461 (92.9%) | <0.001 |
| Anemia | Yes: 452 (3.7) | Yes: 55 (3.8%) | 0.931 |
| Coronary disease | Yes: 1553 (12.7%) | Yes: 265 (18.1%) | <0.001 |
| Cerebrovascular disease | Yes: 183 (1.5%) | Yes: 20 (1.4%) | 0.908 |
| Heart failure | Yes: 1247 (10.2%) | Yes: 221 (15.1%) | <0.001 |
| Rheumatoid arthritis | Yes: 256 (2.1%) | Yes: 21 (1.5%) | 0.251 |
| **Medications** | | | |
| NSAID | Yes: 3291 (26.9%) | Yes: 411 (28.1%) | 0.399 |
| **Labs (IQR)** | | | |
| Triglycerides | 134.6 (102–180) | 133.95 (102–180.12) | 0.412 |
| Hemoglobin | 14.1 (12.9–15.2) | 14 (13–15) | 0.151 |
| Creatinine | 1.18 (1.01–1.37) | 1.24 (1.05–1.47) | <0.001 |
| Potassium | 4.54 (4.24–4.88) | 4.52 (4.23–4.88) | 0.408 |
| HDL cholesterol | 50.3 (41.4–61.5) | 49.95 (40.79–60.1) | 0.016 |
| LDL cholesterol | 103.6 (79.5–129.6) | 102.21 (79.19–127.53) | 0.156 |

**IQR: Interquartile range. NSAID: Non-steroidal anti-inflammatory drugs**

TABLE S3. Sensitivity analysis Model 2

| Variables | Not included  (n=11558) | Included in Model 2  (n=2143) | P-value |
| --- | --- | --- | --- |
| **Sociodemographic** | | | |
| Age | 78 (72–83) | 78 (72–83) | 0.253 |
| Sex | F: 7163 (61.9%) | F: 1249 (58.3%) | 0.001 |
| Stratum | Mid-Level: 10721 (92.8%) | Mid-Level: 1941 (90.6%) | 0.001 |
| Region | Bogotá: 7380 (63.8%) | Bogotá: 1314 (61.3%) | <0.001 |
| Marital status | Unmarried: 6468 (55.9%) | Unmarried: 1162 (54.2%) | 0.306 |
| **Comorbidities** | | | |
| Initial stage | G3a: 8407 (72.7%) | G3a: 1267 (59.1 %) | <0.001 |
|  | G3b: 2428 (21.0%) | G3b: 644 (30.1%) | <0.001 |
|  | G4: 635 (5.6%) | G4: 203 (9.4%) | <0.001 |
|  | G5: 88 (0.7%) | G5: 29 (1.4%) | <0.001 |
| Diabetes | Yes: 2503 (21.6%) | Yes: 404 (18.8%) | 0.003 |
| Hypertension | Yes: 9549 (82.6%) | Yes: 1662 (77.5%) | <0.001 |
| Anemia | Yes: 451 (3.9%) | Yes: 67 (3.1%) | 0.095 |
| Coronary disease | Yes: 1519 (13.1%) | Yes: 296 (13.8%) | 0.420 |
| Cerebrovascular disease | Yes: 179 (1.5%) | Yes: 26 (1.2%) | 0.281 |
| Heart failure | Yes: 1199 (10.4%) | Yes: 266 (12.4%) | 0.005 |
| Rheumatoid arthritis | Yes: 243 (2.1%) | Yes: 26 (1.2%) | 0.008 |
| **Medications** | | | |
| NSAIDs | Yes: 3232 (27.9%) | Yes: 472 (22.1%) | <0.001 |
| **Labs (IQR)** | | | |
| Triglycerides | 135 (102.8–180.7) | 131.8 (99.7–178) | 0.006 |
| Hemoglobin | 14.1 (12.9–15.2) | 13.9 (12.8–15) | <0.001 |
| Creatinine | 1.17 (1.01–1.36) | 1.25 (1.06–1.52) | <0.001 |
| Potassium | 4.54 (4.24–4.88) | 4.54 (4.24–4.90) | 0.176 |
| HDL cholesterol | 50.4 (41.5–61.6) | 49.7 (40.7–60.2) | 0.001 |
| LDL cholesterol | 103.7 (79.7–129.8) | 101.9 (78.2–127.7) | 0.008 |

**IQR: Interquartile range. NSAID: Non-steroidal anti-inflammatory drugs**

Figure S1. External Validation Cohort Selection Process.


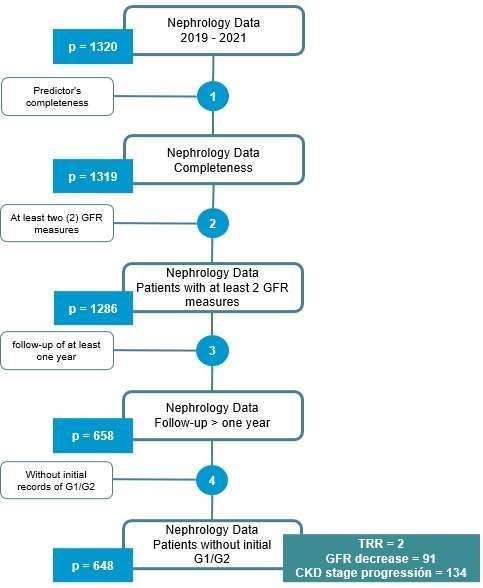


**Table S4. Description of the variables in model 3 in the external validation cohort.**

| **Predictor** | **All** | **Stage Progression** | **Decrease in eGFR** | **RRT** |
| --- | --- | --- | --- | --- |
|  | **N = 648**  **N (%)** | **n = 134**  **N (%)** | **n = 91**  **N (%)** | **n = 2**  **N (%)** |
| **Sex** (%F) | 235 (36.3) | 41 (30.6) | 32 (35.2) | 1 (50.0) |
| **Region**  Bogotá % | 524 (80.9) | 110 (82.1) | 74 (81.3) | 1 (50.0) |
| **Age (years)*** | 77 (68 - 84) | 76 (67 - 82) | 77 (66 - 83) | 51.5 (36 – 67) |
| **Lab tests** |  |  |  |  |
| eGFR mL/min/1.73 m^2^ | 34.5 (26.3 – 42.3) | 34.9 (30.3 – 43.0) | 31.8 (22.1 – 40.6) | 22.4 (19.3 - 25.5) |
| Hemoglobin gr/dL | 14.5 (13 – 16.1) | 14.1 (12.6 – 15.5) | 13.9 (12.4 – 15.3) | 11.8 (10.8 – 12.9) |
| Creatinine mg/dL | 1.6 (1.4 – 2.0) | 1.7 (1.5 – 2.2) | 1.9 (1.5 – 2.4) | 3.8 (2.7 – 4.9) |
| Cholesterol HDL mg/dL | 41.9 (35.0 – 51.7) | 41.5 (35.1 – 50.5) | 41.4 (34 – 47.9) | 44.9 (33.1 – 56.7) |
| Cholesterol LDL mg/dL | 90.5 (67.2 – 119.7) | 89.7 (70.0 – 112.6) | 90.4 (70.8 – 112.7) | 64.4 (49.5 – 79.4) |
| **Comorbidities** |  |  |  |  |
| Diabetes | 216 (33.3) | 54 (40.3) | 41 (45.1) | 1 (50.0) |
| Hypertension | 575 (88.7) | 121 (90.3) | 81 (89.0) | 1 (50.0) |

*Median (IQR)

Table S5. Unadjusted association between predictors and RRT for model 1.

| **Variable** | **Total = 1466** | **With RRT** | **Without RRT** | **OR (CI 95%)** |
| --- | --- | --- | --- | --- |
| **Initial Stage** |  |  |  |  |
| G3a | 0.6111 | 0.0023 | 0.6088 | 0.03 (0.01-0.10) |
| G3b | 0.2981 | 0.0094 | 0.2886 | 0.63 (0.29-1.23) |
| G4 | 0.0804 | 0.0228 | 0.0575 | 16.66 (9.01-30.9) |
| G5 | 0.0102 | 0.0094 | 0.0007 | 324.22 (46.01-12708.01) |
| **Gender (% Fem)** | 0.5970 | 0.0189 | 0.5781 | 0.49 (0.27-0.87) |
| **Stratum** |  |  |  |  |
| Low (1–2) % | 0.0442 | 0.0008 | 0.0433 | 0.38 (0.01-2.31) |
| Mid-Level (3–4) % | 0.9061 | 0.0417 | 0.8612 | 1.94 (0.61-9.85) |
| High (5–6) % | 0.0497 | 0.0015 | 0.0481 | 0.69 (0.08-2.76) |
| **Region** |  |  |  |  |
| Bogotá % | 0.6364 | 0.0315 | 0.6048 | 1.45 (0.78-2.80) |
| Eastern % | 0.0552 | 0.0047 | 0.0504 | 2.15 (0.72-5.28) |
| Central % | 0.1317 | 0.0031 | 0.1285 | 0.49 (0.12-1.37) |
| Pacific % | 0.1136 | 0.0015 | 0.1119 | 0.27 (0.03-1.07) |
| Caribbean % | 0.0623 | 0.0031 | 0.0591 | 1.16 (0.29-3.29) |
| Other | 0.0008 | 0 | 0.0008 | 0 |
| **Marital status**  **(Married %)** | 0.4535 | 0.0228 | 0.4305 | 1.30 (0.73-2.32) |
| **Lab tests** |  |  |  |  |
| Triglycerides | 0.3919 | 0.0252 | 0.3667 | 2.14 (1.20-3.84) |
| *(< 150 mg/dL)* |  |  |  |  |
| Hemoglobin | 0.2176 | 0.0221 | 0.1956 | 3.88 (2.17-6.93) |
| *(F: < 12 g/dL)* |  |  |  |  |
| *(M: < 14 g/dL)* |  |  |  |  |
| Creatinine | 0.8967 | 0.0441 | 0.8525 | ~∞+ (1.75- ∞+) |
| *(M: 0,67–1,17)* |  |  |  |  |
| *(F: 0,51–0,95)* |  |  |  |  |
| Potassium | 0.1617 | 0.0197 | 0.1419 | 4.61 (2.54-8.28) |
| *(3,5–5,1 mEq/L)* |  |  |  |  |
| HDL Cholesterol | 0.2263 | 0.0157 | 0.2106 | 1.96 (1.05-3.55) |
| *(< 40 mg/dL)* |  |  |  |  |
| LDL Cholesterol | 0.4692 | 0.0221 | 0.5087 | 0.87 (0.49-1.56) |
| *(< 100 mg/dL)* |  |  |  |  |
| **Comorbidities** |  |  |  |  |
| Diabetes | 0.2468 | 0.0197 | 0.2271 | 2.58 (1.43-4.60) |
| Hypertension | 0.9290 | 0.0331 | 0.8958 | 0.20 (0.10-0.41) |
| Anemia | 0.0385 | 0.0015 | 0.0370 | 0.91 (0.10-3.67) |
| Coronary disease | 0.1814 | 0.0071 | 0.1742 | 0.85 (0.36-1.80) |
| Cerebrovascular | 0.0142 | 0 | 0.0141 | 0 |
| disease |  |  |  |  |
| Heart failure | 0.1506 | 0.0055 | 0.1451 | 0.79 (0.30-1.80) |
| Rheumatoid | 0.0149 | 0 | 0.0149 | 0 |
| arthritis |  |  |  |  |
| Consumption of | 0.2808 | 0.0078 | 0.2728 | 0.54 (0.24-1.10) |
| NSAIDs (yes) |  |  |  |  |

**Fem: Feminine. NSAID: Non-steroidal anti-inflammatory drugs**

**TABLE S6. Unadjusted association between predictors and decrease in GFR for model 1.**

| **Variable** | **Total = 2143** | **Decrease in**  **GFR** | **No Decrease in**  **GFR** | **OR (CI 95%)** |
| --- | --- | --- | --- | --- |
| **Initial Stage** |  |  |  |  |
| G3a | 0.5912 | 0.1847 | 0.4064 | 0.43 (0.32-0.57) |
| G3b | 0.3005 | 0.1236 | 0.1768 | 1.27 (0.94-1.71) |
| G4 | 0.0947 | 0.0555 | 0.0391 | 3.48 (2.24-5.40) |
| G5 | 0.0135 | 0.0102 | 0.0032 | 12.84 (3.27-73.15) |
| **Sex (%F)** | 0.5828 | 0.1913 | 0.3915 | 0.64 (0.48-0.84) |
| **Stratum** |  |  |  |  |
| Low (1–2) % | 0.0438 | 0.0191 | 0.0247 | 1.01 (0.48-1.99) |
| Mid-Level (3–4) % | 0.9087 | 0.3341 | 0.5749 | 0.67 (0.43-1.05) |
| High (5–6) % | 0.0471 | 0.0195 | 0.0275 | 1.65 (0.89-2.95) |
| **Region** |  |  |  |  |
| Bogotá % | 0.6131 | 0.2449 | 0.3681 | 1.09 (0.81-1.47) |
| Eastern % | 0.0634 | 0.0238 | 0.0396 | 1.31 (0.70-2.32) |
| Central % | 0.1549 | 0.0368 | 0.1181 | 0.38 (0.21-0.65) |
| Pacific % | 0.1129 | 0.0494 | 0.0634 | 1.57 (1.03-2.35) |
| Caribbean % | 0.0546 | 0.0186 | 0.0359 | 1.02 (0.55-1.81) |
| Other | 0.0009 | 0.0004 | 0.0005 | 0 |
| **Marital status (married %)** | 0.4489 | 0.1601 | 0.2888 | 0.81 (0.61-1.08) |
| **Lab tests** |  |  |  |  |
| Triglycerides | 0.3784 | 0.1521 | 0.2263 | 1.06 (0.79-1.40) |
| *(>150 mg/dL)* |  |  |  |  |
| Hemoglobin | 0.2435 | 0.1185 | 0.1250 | 1.74 (1.26-2.38) |
| *(F: < 12 g/dL)* |  |  |  |  |
| *(M: < 14 g/dL)* |  |  |  |  |
| Creatinine | 0.8847 | 0.3378 | 0.5468 | 1.45 (0.88-2.49) |
| *(M: 0,67–1,17)* |  |  |  |  |
| *(F: 0,51–0,95)* |  |  |  |  |
| Potassium | 0.1684 | 0.0779 | 0.0905 | 1.55 (1.08-2.20) |
| *(3,5–5,1 mEq/L)* |  |  |  |  |
| HDL Cholesterol | 0.2281 | 0.1022 | 0.1259 | 1.37 (0.99-1.88) |
| *(> 40 mg/dL)* |  |  |  |  |
| LDL Cholesterol | 0.5254 | 0.1819 | 0.3434 | 0.74 (0.56-0.99) |
| *(< 100 mg/dL)* |  |  |  |  |
| **Comorbidities** |  |  |  |  |
| Diabetes | 0.1885 | 0.0933 | 0.0951 | 2.05 (1.51-2.77) |
| Hypertension | 0.7755 | 0.2781 | 0.4974 | 0.40 (0.25-0.66) |
| Anemia | 0.0312 | 0.0135 | 0.0177 | 1.51 (0.74-2.94) |
| Coronary disease | 0.1381 | 0.0671 | 0.0709 | 1.40 (0.99-1.98) |
| Cerebrovascular | 0.0121 | 0.0037 | 0.0084 | 0.74 (0.13-2.65) |
| disease |  |  |  |  |
| Heart failure | 0.1241 | 0.0746 | 0.0495 | 1.58 (1.09-2.27) |
| Rheumatoid arthritis | 0.0121 | 0.0028 | 0.0093 | 0.69 (0.12-2.45) |
| Consumption of | 0.2202 | 0.0718 | 0.1484 | 0.59 (0.42-0.83) |
| NSAIDs (YES) |  |  |  |  |

**TABLE S7. Unadjusted association between each predictor and RRT for model 2.**

| **Predictor** | **Total = 2143** | **With RRT** | **Without TRR** | **OR (CI 95%)** |
| --- | --- | --- | --- | --- |
| **Initial Stage** |  |  |  |  |
| G3a | 0.5912 | 0.0028 | 0.5884 | 0.03 (0.02- 0.04) |
| G3b | 0.3005 | 0.0084 | 0.2921 | 0.54 (0.49- 0.60) |
| G4 | 0.0947 | 0.0191 | 0.0756 | 8.78 (8.05- 9.57) |
| G5 | 0.0135 | 0.0083 | 0.0051 | 57.48 (47.82- 69.34) |
| **Sex (% Fem)** | 0.5828 | 0.0172 | 0.5656 | 0.65 (0.60- 0.71) |
| **Stratum** |  |  |  |  |
| Low (1–2) % | 0.0438 | 0.0004 | 0.0434 | 0.12 (0.07- 0.21) |
| Mid-Level (3–4) % | 0.9087 | 0.0368 | 0.8689 | 1.69 (1.42- 2.02) |
| High (5–6) % | 0.0471 | 0.0014 | 0.0457 | 1.09 (0.90- 1.31) |
| **Region** |  |  |  |  |
| Bogotá % | 0.6131 | 0.0256 | 0.5874 | 1.31 (1.19- 1.43) |
| Eastern % | 0.0634 | 0.0037 | 0.0597 | 2.22 (1.95- 2.52) |
| Central % | 0.1549 | 0.0046 | 0.1502 | 0.54 (0.46- 0.64) |
| Pacific % | 0.1129 | 0.0023 | 0.1106 | 0.40 (0.33- 0.48) |
| Caribbean % | 0.0546 | 0.0023 | 0.9613 | 0.70 (0.56- 0.86) |
| Other | 0.0009 | 0 | 0.0009 | 0 |
| **Marital status (%**  **Married)** | 0.4489 | 0.0214 | 0.4274 | 1.49 (1.37- 1.62) |
| **Lab tests** |  |  |  |  |
| Triglycerides | 0.3784 | 0.0191 | 0.3593 | 1.71 (1.57- 1.85) |
| Hemoglobin | 0.2435 | 0.0177 | 0.2258 | 2.56 (2.36- 2.78) |
| Creatinine | 0.8847 | 0.0382 | 0.8464 | 237.20 (42.51- 8824.07) |
| Potassium | 0.1684 | 0.0168 | 0.1516 | 4.17 (3.84- 4.54) |
| HDL cholesterol | 0.2281 | 0.0121 | 0.2160 | 1.62 (1.49- 1.77) |
| LDL cholesterol | 0.5254 | 0.0201 | 0.5053 | 0.92 (0.85- 1.00) |
| **Comorbidities** |  |  |  |  |
| Diabetes | 0.1885 | 0.0135 | 0.1749 | 1.77 (1.63- 1.93) |
| Hypertension | 0.7755 | 0.0261 | 0.7494 | 0.37 (0.34- 0.40) |
| Anemia | 0.0312 | 0.0009 | 0.0303 | 0.52 (0.39- 0.68) |
| Coronary disease | 0.1381 | 0.0056 | 0.1325 | 0.57 (0.51- 0.64) |
| Cerebrovascular | 0.0121 | 0.0005 | 0.0116 | 0.35 (0.20- 0.58) |
| disease |  |  |  |  |
| Heart failure | 0.1241 | 0.0046 | 0.1195 | 0.53 (0.46- 0.59) |
| Rheumatoid arthritis | 0.0121 | 0 | 0.0121 | 0 |
| Consumption of | 0.2202 | 0.0051 | 0.2151 | 0.45 (0.40- 0.51) |
| NSAIDs (YES) |  |  |  |  |

**Fem: Feminine. NSAID: Non-steroidal anti-inflammatory drugs**

TABLE S8. Unadjusted association between predictor and decrease in GFR for model 2.

| **Variable** | **Total = 2143** | **Decrease in GFR** | **No Decrease in GFR** | **OR (CI 95%)** |
| --- | --- | --- | --- | --- |
| **Initial Stage** |  |  |  |  |
| G3a | 0.5912 | 0.1847 | 0.4064 | 0.57 (0.54- 0.61) |
| G3b | 0.3005 | 0.1236 | 0.1768 | 0.90 (0.84- 0.96) |
| G4 | 0.0947 | 0.0555 | 0.0391 | 2.31 (2.14- 2.49) |
| G5 | 0.0135 | 0.0102 | 0.0032 | 3.45 (2.97- 4.02) |
| **Gender** (% Fem) | 0.5828 | 0.1913 | 0.3915 | 0.68 (0.64- 0.72) |
| **Stratum** |  |  |  |  |
| Low (1–2) % | 0.0438 | 0.0191 | 0.0247 | 0.74 (0.62- 0.87) |
| Mid-Level (3–4) % | 0.9087 | 0.3341 | 0.5749 | 1.31 (1.16- 1.47) |
| High (5–6) % | 0.0471 | 0.0195 | 0.0275 | 0.77 (0.66- 0.91) |
| **Region** |  |  |  |  |
| Bogotá % | 0.6131 | 0.2449 | 0.3681 | 1.42 (1.33- 1.52) |
| Eastern % | 0.0634 | 0.0238 | 0.0396 | 0.93 (0.82- 1.05) |
| Central % | 0.1549 | 0.0368 | 0.1181 | 0.54 (0.47- 0.61) |
| Pacific % | 0.1129 | 0.0494 | 0.0634 | 0.90 (0.81- 0.99) |
| Caribbean % | 0.0546 | 0.0186 | 0.0359 | 0.72 (0.61- 0.84) |
| Other | 0.0009 | 0.0004 | 0.0005 | 0.58 (0.06- 2.43) |
| **Marital status (% Married)** | 0.4489 | 0.1601 | 0.2888 | 1.06 (1.00- 1.13) |
| **Lab tests** |  |  |  |  |
| Triglycerides | 0.3784 | 0.1521 | 0.2263 | 1.34 (1.26- 1.43) |
| *(>150 mg/dL)* |  |  |  |  |
| Hemoglobin | 0.2435 | 0.1185 | 0.1250 | 1.51 (1.41- 1.61) |
| *(F: < 12 g/dL)* |  |  |  |  |
| *(M: < 14 g/dL)* |  |  |  |  |
| Creatinine | 0.8847 | 0.3378 | 0.5468 | 1.74 (1.52- 2.00) |
| *(M: 0,67–1,17)* |  |  |  |  |
| *(F: 0,51–0,95)* |  |  |  |  |
| Potassium | 0.1684 | 0.0779 | 0.0905 | 1.47 (1.37- 1.58) |
| *(3,5–5,1 mEq/L)* |  |  |  |  |
| HDL Cholesterol | 0.2281 | 0.1022 | 0.1259 | 1.53 (1.43- 1.63) |
| *(> 40 mg/dL)* |  |  |  |  |
| LDL Cholesterol | 0.5254 | 0.1819 | 0.3434 | 0.97 (0.91- 1.03) |
| *(< 100 mg/dL)* |  |  |  |  |
| **Comorbidities** |  |  |  |  |
| Diabetes | 0.1885 | 0.0933 | 0.0951 | 1.44 (1.35- 1.54) |
| Hypertension | 0.7755 | 0.2781 | 0.4974 | 0.49 (0.46- 0.53) |
| Anemia | 0.0312 | 0.0135 | 0.0177 | 1.11 (0.95- 1.28) |
| Coronary disease | 0.1381 | 0.0671 | 0.0709 | 1.07 (1.00- 1.16) |
| Cerebrovascular | 0.0121 | 0.0037 | 0.0084 | 0.43 (0.30- 0.59) |
| disease |  |  |  |  |
| Heart failure | 0.1241 | 0.0746 | 0.0495 | 1.20 (1.12- 1.29) |
| Rheumatoid arthritis | 0.0121 | 0.0028 | 0.0093 | 0.82 (0.65- 1.04) |
| Consumption of | 0.2202 | 0.0718 | 0.1484 | 0.64 (0.59- 0.69) |
| NSAID (% YES) |  |  |  |  |

**Fem: Feminine**. **NSAID: Non-steroidal anti-inflammatory drugs. GFR: Glomerular Filtration Rate**

**Figure S2. Calibration plots for Model 1**

**
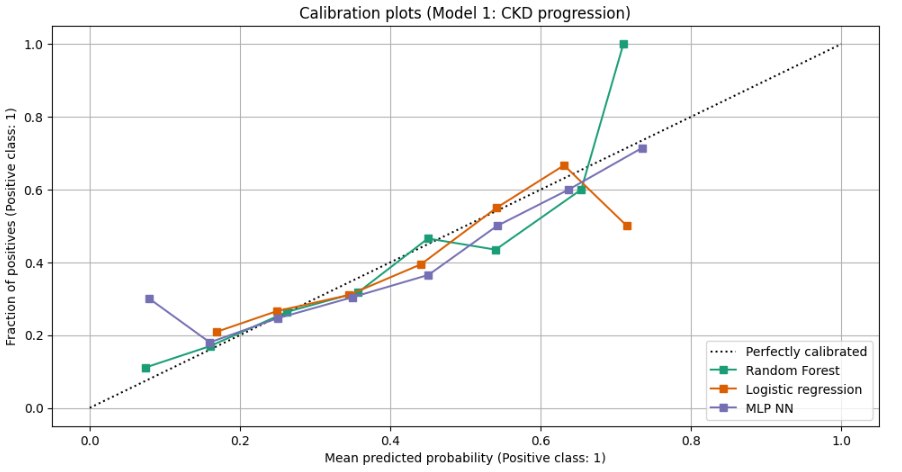

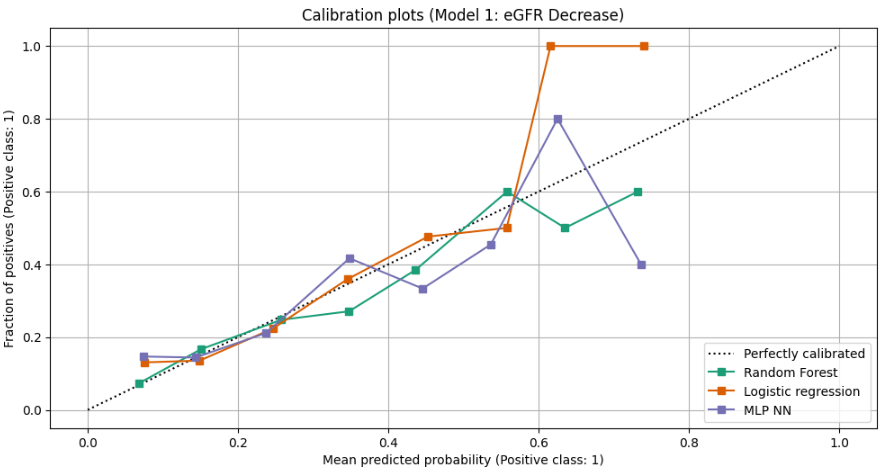

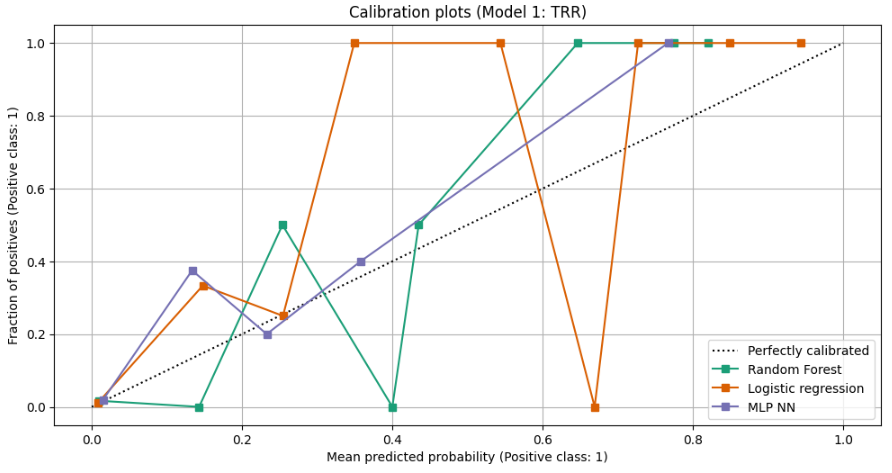
**
